# Why Don’t I Help You? The Relationship between Role Stressors and Helping Behavior from a Cognitive Dissonance Perspective

**DOI:** 10.3389/fpsyg.2017.02220

**Published:** 2018-01-24

**Authors:** Li Zhang, Ying Xia, Baowei Liu, Lu Han

**Affiliations:** ^1^School of Business, Harbin Institute of Technology, Harbin, China; ^2^Beijing Peony Electronic Group Co., Ltd., Beijing, China

**Keywords:** role stressors, cognitive dissonance, normative commitment, helping behavior, perceived organizational support

## Abstract

This paper proposes that role stressors decrease helping behavior by undermining employees’ normative commitment from a cognitive dissonance perspective and social exchange theory. We also propose two competitive assumptions of the moderating effect of perceived organizational support (POS). In this paper, we first examine these hypotheses in Study 1 and then verify the cognitive dissonance perspective in Study 2. In Study 1, we collected data from 350 employees of two enterprises in China. The results indicated that role stressors had a negative link with helping behavior via the mediating role of normative commitment. The results also showed that POS strengthened the negative relationship between role stressors and normative commitment. In Study 2, we invited 104 employees to participate in a scenario experiment. The results found that role stressors had an impact on normative commitment via dissonance. Our studies verified the combination of cognitive dissonance perspective and social exchange theory to explain the impact of role stressors on helping behavior.

## Introduction

The association between role stressors and employees’ performance has been demonstrated in numerous studies over the past 40 years (Jackson and Schuler, 1985). In recent years, more and more researchers have investigated its influence on extra-role performance such as organizational citizenship behavior (OCB) (Eatough et al., 2011). Owning to its discretionary nature, OCB is suspected to be more greatly affected by role stressors than in-role behaviors. Following this logic, the present study focuses specifically on helping behavior, defined as “actions that one person takes to assist colleagues or benefit the organization as a whole" (Sparrowe et al., 2006, p. 1194), because it has more discretion than many other forms of extra-role behaviors (Jex et al., 2003; Organ et al., 2006). Many scholars have also studied helping as an independent construct (Kacmar et al., 2013; Hirst et al., 2016) because it has a stable and critical influence on workgroup effectiveness (Huffmeier and Hertel, 2011).

Scholars have tried to unpack the relationship between role stressors and helping behavior from several different perspectives. From a resources allocation perspective, Jex et al. (2003) report that experiencing role stressors forces employees to allocate more resources to their in-role tasks and pay less attention to extra-role behaviors. From an emotional perspective, Jackson and Schuler (1985) find that role stressors give rise to experiences of negative emotions such as anxiety and tension, which are negatively related to prosocial behaviors like helping (Samnani et al., 2014). In addition, from a social exchange perspective, employees exposed to role stressors are likely to experience a decrease in general job satisfaction and thus being less likely to engage in extra-role behavior (Eatough et al., 2011).

All these perspectives can explain the relationship between role stressors and helping behavior; however, they have all considered role stressors as one random type of job stressors from exchange, resource, and emotion perspectives in general, which have failed to capture the unique feature of role stressors. Actually, a role stressor can be a particular job stressor that can influence cognition directly as it involves one’s own perception and the role sender’s expectations (Barling et al., 2004). Such perception influences employees’ cognition (Fred Miao and Evans, 2007) and induces the cognitive dissonance process. Prior studies argued that the cognition component is a more powerful determinant of OCB than mood state (Organ and Konovsky, 1989); that is to say, a good cognitive appraisal of the job would drive employees to engage in more OCBs.

Our research aims to explain the impact of role stressors on helping by combining the cognitive dissonance perspective and social exchange theory. Cognitive dissonance perspective means that individuals with inconsistent cognitions experience dissonance (unpleasant state), and they are motivated to reduce dissonance by altering cognition (Festinger, 1962). When employees are exposed to role stressors, they are likely to experience dissonance because their role perception is different from their expectation. The dissonance will lead to negative exchange relationship between the individual and the organization. That is, when employees are exposed to role stressors, they will experience feelings of dissonance, which decreases their normative commitment and then helping behavior.

Furthermore, we endeavor to identify the boundary condition for role stressors—helping behavior relationship. On the one hand, perceived organizational support (POS), which is the indicator that the organization cares about the employee (Eisenberger et al., 1990), has been regarded as an effective buffer for the stress effect following the social exchange theory. On the other hand, cognitive dissonance theory indicates that freedom of choice is necessary for dissonance arousal to occur (Festinger, 1962, 2010; Harmon-Jones and Harmon-Jones, 2008). Following this logic, POS can be viewed as freedom of choice and enhances the negative effect of role stressors. Thus, we propose competitive assumptions of the moderating effect of POS to explore whether it enhances or weakens the influence of role stressors on helping behavior.

**FIGURE 1:**
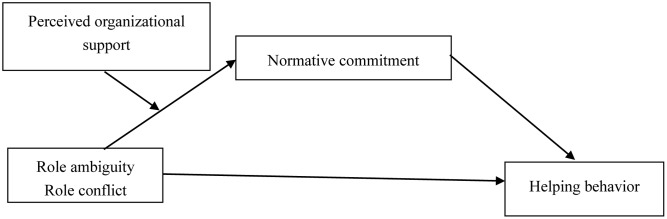
Research model.

The cognitive dissonance process includes four steps: cognition discrepancy occurring, dissonance occurring, being motivated to reduce dissonance, and discrepancy reduction (Hinojosa et al., 2017). Many studies in management area have used cognitive dissonance theory as the theoretical background and included cognitive dissonance in their theoretical framework. Nevertheless, they have not measured dissonance empirically (see Hinojosa et al., 2017, for a review). We try to improve such limitation and try to test the dissonance process empirically. However, we found that most of the studies investigating cognitive dissonance have used the experimental method (e.g., Gino, 2008; Bashshur et al., 2011; Stoverink et al., 2014). Studies have also suggested that experimental manipulation can ensure that psychological state is created under the context (Spencer et al., 2005). Thus, we focus on the dissonance occurring and discrepancy reduction steps with different research methods separately in the following two studies. Study 1 reflects the discrepancy reduction step in the process, and we examine the moderated mediation model (as depicted in **Figure 1**) using data from 350 employees of two enterprises in China. In Study 2, we verify the cognitive dissonance perspective by testing the cognitive dissonance process; specifically, we conduct a scenario experiment to test the mediating role of dissonance in the role ambiguity – normative commitment relationship. We also provide theoretical and practical contributions to the existing literature on role stressors, cognitive dissonance, and helping behavior.

## Study 1

### Role Stressors and Normative Commitment

Role stressors are some of the most commonly studied work stressors (Tubre and Collins, 2000). Several meta-analyses have investigated the influence of role stressors in the organization (Fisher and Gitelson, 1983; Jackson and Schuler, 1985; Örtqvist and Wincent, 2006), and it is reported that role stressors are still big issues that need attention in the current era (Bowling et al., 2017). Thus, our research focuses on role stressors as the independent variable. The present study investigates role stressors consisting of role ambiguity and role conflict, which may be key factors in creating environmental conditions for helping behavior at workplace.

Normative commitment, which indicates employees’ sense of obligation to their organizations, is one of the three types of organizational commitment included in the three-component model developed by Meyer and Allen (1991). In this study, we follow the definition of Wiener (1982) and propose that normative commitment is an individual’s internalized pressure to act for the organization’s goals and interests. We focus on normative commitment (other than other forms of commitment) in this model because it is reasonable to consider the influence of role stressors on organizational commitment following the exchange relationship between the employee and the organization. More importantly, normative commitment indicates the general obligation to the organization, which can reflect the cognitive nature of our theoretical framework. According to the cognitive dissonance theory, individuals experience uncomfortable state (i.e., dissonance) when they have inconsistent cognitions, and such uncomfortable state motivates individuals to change their cognitions. We follow this theoretical framework and predict that normative commitment can reflect employees’ cognition in the cognitive dissonance process.

When exposed to role ambiguity, employees cannot clearly catch the organization’s expectations; even they don’t know what to do to achieve their role tasks. Such perceptions of role ambiguity leave employees in a situation that they don’t know what and how to do to enhance their self-achievement and improve task performance. These perceptions are different from employees’ expectations for their job. Employees with such ambiguous cognitions will experience dissonance, which motivates them to change cognition. When encountering role ambiguity, employees need to find out ways to deal with these stressors and ensure their in-role performance. Their attitude will change to consider their own interest rather than serving the best interests of their organization. To be more specific, employees encountering role ambiguity will experience dissonance, and such uncomfortable feelings inspire negative reciprocity. Thus, employees decrease their normative commitment to the organization by considering the exchange relationship with their organization.

When exposed to role conflict, employees usually perceive different role expectations from their supervisors or from their colleagues (Schmidt et al., 2014). Employees encountering with role conflict have to deal with many temporary arrangements, and these arrangements will disturb their normal tempo and plan. Similar to the influence of role ambiguity, we predict that role conflict will also increase employees’ cognitive dissonance, and such dissonance can induce negative reciprocity with the organization. That is the normative commitment to the organization decreased in our study. Moreover, cognitive dissonance theory suggests that cognition change should be consistent with individual’s recent behavior. For example, a person who believes smoking is unhealthy smoked a bit recently, and then he is likely to change his cognition to that smoking is not very unhealthy. When encountering role conflict, employees need to find ways to deal with these conflict arrangements, and at the same time, they strive to ensure that their in-role performance would not be damaged. Their cognition will focus on the solution to ensure their in-role performance and the upfront difficulties rather than considering the interest for their organization as the cognition change is consistent with their recent behavior. Thus, we propose that employees encountering role conflict will decrease their normative commitment to the organization to maintain consistency in cognition.

Previous studies have also supported the negative impact of role stressors on employee’s normative commitment. According to the study of Yousef (2002), role ambiguity and role conflict are negatively related to normative commitment to the mediating effect of job satisfaction. Addae et al. (2008) also found that role ambiguity and role conflict were negatively related to normative commitment in the public sector.

To sum up, we propose the following hypothesis:

Hypothesis 1a: Role ambiguity has a negative relationship with normative commitment.
Hypothesis 1b: Role conflict has a negative relationship with normative commitment.

### Normative Commitment and Helping Behavior

Helping is a kind of interpersonal, cooperative extra-role behavior that is beneficial to their whole organization (Van Dyne and LePine, 1998). It is different from in-role tasks, which are specified in the job descriptions. Helping is not entailed by job regulations, and it does not bring any punishment if employee undoes that (Kuehn and Al-Busaidi, 2002). Therefore, an employee has very high discretion in deciding whether or not to help others in the organization.

As mentioned earlier, normative commitment is based on personal norms governing how one believes one ought to act (Meyer and Parfyonova, 2010). Thus, it can be expected to influence individual behavior. Normative commitment facilitates helping behavior because employees in high normative commitment tend to feel obligated to their organization, and have a higher sense of “should do” for their organization’s whole interest. Such obligation motivates employees to engage more in helping their coworkers, which can eventually be good for the benefits of the whole organization.

Moreover, the impact of normative commitment on helping behavior can also be attributed to the normative explanation of helping behavior. Such stream of explanation is to regard norm as an attitudinal variable (Fishbein, 1967; Schwartz, 1973). It postulates that individuals recognize a line of behaviors that the norms are perceived to apply (Schwartz, 1973). Empirical evidence indicates that normative commitment is positively related to helping behavior. For example, Meyer et al. (1993) find that normative commitment is positively related to OCBs such as helping others. Morrison (1994) indicates that the higher the level of normative commitment, the more broadly the employee will define their job responsibilities, and the more so-called OCBs they will define as in-role. Jain et al. (2013) found a positive relationship between POS and OCBs, and POS is expected to increase organizational commitment.

Accordingly, we propose:

Hypothesis 2: Normative commitment has a positive relationship with helping behavior.

Empirical evidence has claimed that role ambiguity and role conflict have negative influences on employees’ extra-role behaviors (Eatough et al., 2011). Jex et al. (2003) also report that employees experiencing role ambiguity or role conflict tend to decrease their engagement in helping behavior according to social exchange theory. Moreover, cognitive dissonance research has argued that people tend to change their behavior to be consistent with their beliefs (Aronson et al., 1991; Harmon-Jones and Harmon-Jones, 2007). Thus, based on cognitive dissonance theory and social exchange theory, employees exposed to role ambiguity or role conflict will suffer cognitive dissonance, which causes them to decrease normative commitment and their engagement in helping behavior. As such, we propose:

Hypothesis 3a: Role ambiguity has an indirect relationship with helping behavior through normative commitment.
Hypothesis 3b: Role conflict has an indirect relationship with helping behavior through normative commitment.

### The Moderating Role of Perceived Organization Support

Perceived organizational support provides employees with resources to deal with stressors in the workplace. Although many studies have supported that POS buffers the undesired influence of role stressors following social exchange theory, there is also evidence suggesting that POS strengthens the negative effect of work stressors on OCBs (Jain et al., 2013). Research also suggests that organizational support may enhance negative results when they are unsolicited or become overwhelming (Deelstra et al., 2003). In the current study, we argue that it is possible that role ambiguity and role conflict induce employees’ cognitive dissonance, which decreases their normative commitment. Thus, we try to propose a pair of competitive assumptions to explore the actual moderating effect of POS on the relationship between role stressors and helping behavior based on cognitive dissonance theory and social exchange theory.

On the one hand, we propose that POS weakens the negative impact of role stressors on normative commitment. Support provided by the organization can be solutions for employees to deal with role stressors. That means POS can help to deal with role stressors and buffer the negative effect induced by role stressors. Moreover, employees who receive support from the organization have a good relationship with organization because of the good treatment toward them. Such good attribution will buffer the negative influence caused by role stressors. Thus, we can predict that POS buffers the negative effect of role stressors on normative commitment.

We propose the following:

Hypothesis 4a: POS moderates the relationship between role ambiguity and normative commitment, such that the negative relationship is weaker when employees are higher in POS.
Hypothesis 4b: POS moderates the relationship between role conflict and normative commitment, such that the negative relationship is weaker when employees are higher in POS.

On the other hand, we propose that a high level of POS will strengthen the negative impact of role stressors on normative commitment. Brehm and Cohen (1962) and Festinger (1962) postulated that freedom of choice is necessary for dissonance arousal to occur. Without free choice, an individual would not have any choice to change their attitude to keep consistency (Festinger, 2010). As a result, efforts to maintain cognitive consistency between attitudes are more likely to occur when people perceive themselves as having choices. There are some studies using the freedom of choice logic to propose hypotheses. For example, Brett et al. (1995) regard employees’ financial requirements as freedom of choice to predict its moderating effect on organizational commitment—performance relationship based on cognitive dissonance theory. To be specific, they regard low financial requirements as allowing freedom of choice and high financial requirements as less freedom of choice.

Following this logic, we argue that high POS is regarded as allowing employees’ freedom of choice because POS can provide employees with resources to deal with role stressors. Under high POS condition, employees can believe that their organization is responsible for them as it provides them with useful resources. They think their organization is blamable as for the role ambiguity or conflict they perceive. At the same time, they may perceive that their organization is good as for the resources or solutions it provides. POS spurs these two cognitions to generate and exist in their mind, which causes dissonance. However, when the POS is low, the employees have enough evidence to believe that their organization is blamable. Things are so clear that they don’t need to think hard to understand them. Therefore, POS can be an enhancer of dissonance producing. We expect there would be a greater negative relationship between role stressors and normative commitment for employees with high POS than for employees with low POS.

Thus, we propose the following:

Hypothesis 5a: POS moderates the relationship between role ambiguity and normative commitment, such that the negative relationship is stronger when employees are higher in POS.
Hypothesis 5b: POS moderates the relationship between role conflict and normative commitment, such that the negative relationship is stronger when employees are higher in POS.

Overall, we propose that role ambiguity and role conflict will cause employees’ attitude change to the organization—decrease in normative commitment—and decrease the helping behavior of these employees. We propose that such attitude change influence is more salient or weaker for those with higher POS. Thus, we propose the following hypothesis to represents a first-stage moderated mediation model:

Hypothesis 6a: POS moderates the mediation effect of normative commitment on the relationship between role ambiguity and helping behavior, such that the indirect effect is stronger (weaker) when employees are higher in POS.
Hypothesis 6b: POS moderates the mediation effect of normative commitment on the relationship between role conflict and helping behavior, such that the indirect effect is stronger (weaker) when employees are higher in POS.

### Method

#### Participants

We collected data from two manufacturing companies in northeast China. We got a randomly selected list of 400 employees with the assistance of human resources management department. All of the employees ensured that they participated in the survey process voluntarily, and we got their written informed consent before participation. The study procedures were approved by the local ethics committee and were in line with the 1964 Helsinki Declaration.

We distributed 400 questionnaires to the participants in the morning of the workday, and the human resources management department helped us to describe the principles and tips of the investigation and to remind employees to answer the questionnaires during the workday. We collected the questionnaires with the help of the human resources managers at the end of the workday. We received 350 valid questionnaires at last, with a response rate of 87.5%. Of the 350 employees, 63% were men, 37% were women. In terms of age, 45.1% were aged 29 or below, 31.2% were between 30 and 39 years old, 18.8% were aged between 40 and 49 years old, and 4.9% were aged 50 or above. Regarding tenure, 58% of respondents had less than 5 years of job experience and 16.9% of respondents had 5–10 years of job experience. In terms of education, 18% held junior college degrees, 70% held a bachelor’s degree, and 10% held a master’s or higher degree. Regarding job tasks, 56.9% were engineers or technical personnel; 5.4% were marketing specialists; 20.3% were in the managerial position; 6.9% of employees were working in the financial department; 4.3% were in the production department, and 6.3% were doing back service or other types of job.

#### Measurements

We used a translation – back translation procedure to ensure accuracy of the questionnaire (Brislin, 1980). We established a scale including role stressor, normative commitment, POS, helping behavior, and control variables. All measures use the 5-Likert scale, ranging from 1 *(strongly disagree)* to 5 *(strongly agree).*

##### Role stressor

We measured role stressors using the 14-item scale developed by Rizzo et al. (1970). This scale contains six items referring to role ambiguity (e.g., I feel certain about how much authority I have) and eight items referring to role conflict (e.g., I have to do things that should be done differently). Specifically, items of role ambiguity are inversed in meaning. Cronbach’s alpha of role ambiguity and role conflict was 0.81 and 0.84, respectively.

##### Normative commitment

We measured normative commitment based on the scale developed by Meyer et al. (1993) because it has advantages in reflecting the cognitive essence of normative commitment. We adapted this scale to four items in accordance with Bansal et al. (2004). We deleted the reverse-scored item “I do not feel any obligation to remain in the organization” because the reverse-scored item tends to cause inconsistency in factor analysis (Schmitt and Stults, 1986; Idaszak and Drasgow, 1987). We also deleted the item “I believe people who have been trained in a profession have a responsibility to stay in that profession for a reasonable period of time” because the meaning will be different if we simply change the expression of “profession” to “organization.” Cronbach’s alpha of this four-item scale was 0.86.

##### Helping behavior

Following Podsakoff et al. (1990), we measured helping behavior with a five-item scale. The participants were asked to assess to what extent they help their co-workers. A sample item was “I am willing to help others who have work-related problems.” Cronbach’s alpha was 0.92.

##### Perceived organizational support

A four-item scale developed by Hayton et al. (2012) was used. A sample item was “The organization is willing to help me when I need a special favor.” Cronbach’s alpha was 0.82.

##### Control variables

We controlled for organization effect and employees’ gender and education because of their potential effects on employee behavior. A dummy variable was created to represent the two organizations in our sample. Male respondents were dummy-coded as “0” and female respondents were dummy-coded as “1.” Education was coded as “1” for employees who finished high school or below, “2” for employees who held junior college degrees, “3” for employees who held bachelor’s degrees, and “4” for employees who held master’s degrees or higher. In addition, we controlled for tenure (in years) because past research has demonstrated its influence on Chinese employees’ work perceptions and attitudes (Hui and Tan, 1995).

### Results

#### Confirmatory Factor Analyses

Confirmatory factor analysis (CFA) was conducted to examine the construct validity. We created item parcels for role ambiguity and role conflict by using the single-factor method, which is the most frequently used method as indicated by Landis et al. (2000). The hypothesized five-factor measurement model (consisting of role ambiguity, role conflict, normative commitment, helping behavior, and perceived organizational support) showed a better fit to our data (CFI = 0.91, GFI = 0.89, RMSEA = 0.09, χ^2^ = 399.56, *df* = 94) than alternative models.

#### Hypotheses Tests

**Table 1** presents the means, standard deviations, and correlation coefficients of variables. Consistent with our arguments, role ambiguity was negatively associated with normative commitment (*r* = -0.51, *p* < 0.01) and helping behavior (*r* = -0.40, *p* < 0.01); role conflict was negatively associated with normative commitment (*r* = -0.14, *p* < 0.01) and helping behavior (*r* = -0.01, *p* > 0.1); normative commitment was positively associated with helping behavior (*r* = 0.63, *p* < 0.01). These results provide preliminary evidence to support our hypotheses.

**Table 1:** Descriptive statistics, correlations of variables in Study 1.

	Mean	*SD*	1	2	3	4	5	6	7	8	9
(1) Company^a^	0.47	0.49									
(2) Gender^b^	1.37	0.48	-0.11^∗^								
(4) Education^c^	2.90	0.56	0.23^∗∗^	0.01	-0.27^∗∗^						
(5) Tenure	7.86	7.99	-0.25^∗∗^	0.07	0.79^∗∗^	-0.23^∗∗^					
(6) Role ambiguity	2.41	0.56	0.11^∗^	0.08	-0.12^∗^	0.18^∗∗^	-0.02				
(7) Role conflict	3.22	0.61	0.32^∗∗^	-0.11^∗^	-0.07	0.06	-0.03	0.22^∗∗^			
(8) Normative commitment	3.61	0.59	-0.05	-0.12^∗^	0.08	0.01	-0.01	-0.51^∗∗^	-0.14^∗∗^		
(9) Perceived organizational support	3.38	0.68	-0.06	-0.08	0.13^∗^	-0.05	0.01	-0.55^∗∗^	0.32^∗∗^	0.44^∗∗^	
(10) Helping behavior	3.79	0.61	0.01	0.01	0.12^∗^	0.01	-0.02	-0.40^∗∗^	-0.01	0.63^∗∗^	0.43^∗∗^

*n = 350; ^a^Company: 0 = Company A; 1 = Company B; ^b^Gender: 0 = Male; 1 = Female; ^c^Education: 1 = high school or below; 2 = junior college degree; 3 = bachelor’s degree; and 4 = master’s degree or higher; ^∗^*p* < 0.05, ^∗∗^*p* < 0.01*.

Hierarchical regression analysis was adopted to test Hypotheses 1–4. Results are shown in **Table 2**. Hypothesis 1a, which predicted a negative relationship between role ambiguity and normative commitment, was supported (β = -0.51, *p* < 0.001, Model 2 in **Table 2**). However, the negative relationship between role conflict and normative commitment was not significant (β = -0.05, *p* > 0.1, Model 2 in **Table 2**). Thus, Hypothesis 1b was not supported. We did not test the mediating role of normative commitment on the role conflict–helping relationship (H3b) further. Results also supported a positive direct link between normative commitment and helping behavior (Hypothesis 2, β = 0.59, *p* < 0.001, Model 6 in **Table 2**).

**Table 2:** Summary of regression analysis results in Study 1.

	Normative commitment			Helping behavior		
	Model 1	Model 2	Model 3	Model 4	Model 5	Model 6
**Control variables**						
Company^a^	-0.08	-0.02	-0.02	0.01	0.02	0.03
Gender^b^	-0.14^∗^	-0.10^∗^	-0.07^†^	-0.00	0.05	0.11^∗^
Education^c^	0.03	0.11^∗^	0.11^∗^	-0.01	0.06	0.00
Tenure	-0.01	0.01	0.02	-0.02	-0.02	-0.02^∗∗^
**Independent variable**						
Role ambiguity		-0.51^∗∗∗^	-0.09		-0.44^∗∗∗^	-0.13^∗^
Role conflict		-0.05	0.65		0.09	0.12^∗∗^
**Moderator**						
Perceived organizational support			1.13^∗∗∗^			
**Interaction**						
Role ambiguity ^∗^ POS			-0.29^†^			
Role conflict ^∗^ POS			-0.76^∗∗^			
**Mediator**						
Normative commitment						0.59^∗∗∗^
*R*^2^	0.02	0.27	0.36	0.00	0.17	0.43
ΔR^2^		0.25^∗∗∗^	0.09^∗∗∗^		0.17^∗∗∗^	0.25^∗∗∗^

*n = 350; ^a^Company: 0 = Company A; 1 = Company B; ^b^Gender: 0 = Male; 1 = Female; ^c^Education: 1 = high school or below; 2 = junior college degree; 3 = bachelor’s degree; and 4 = master’s degree or higher; ^†^*p* < 0.1, ^∗^*p* < 0.05, ^∗∗^*p* < 0.01, ^∗∗∗^*p* < 0.001*.

Hypothesis 3a, which predicted the indirect relationship of role ambiguity and helping through normative commitment, was also supported. The direct path from role ambiguity to helping behavior became less significant when normative commitment was included in the regression model (β = -0.13, *p* < 0.05, Model 6 in **Table 2**), and explained an additional 25% of the variance. We also estimated their 95% confidence intervals following the program of Preacher and Hayes (2008). Results show that the indirect relationship through normative commitment was significant (indirect effect = -0.33, *SE* = 0.046, 95% C.I. = -0.427 to -0.251). Overall, H3a was supported.

Next, we tested the moderating role of POS. **Table 2** shows the regression results for Hypothesis 4a (5a) and 4b (5b). As shown in Model 3, the interaction of role ambiguity and POS was negatively but not significantly related to normative commitment (β = -0.29, *p* > 0.05, Model 3 in **Table 2**). Thus, the moderating effect of POS on role ambiguity and normative commitment was not significant. Hypothesis 5a was not supported.

**FIGURE 2:**
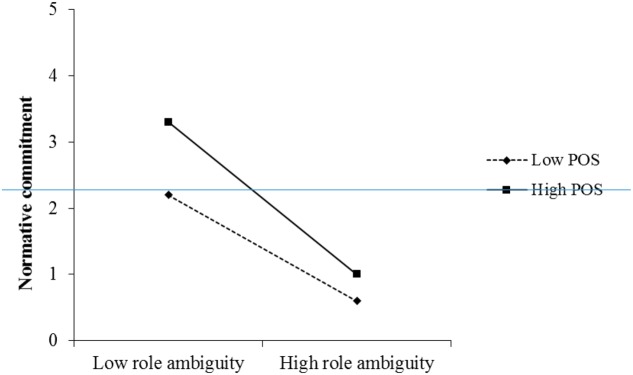
The moderating effect of perceived organizational support on the relationship between role conflict and normative commitment.

Moreover, the moderating effect of POS on the role conflict–normative commitment relationship was significant as the interaction of role conflict and POS was negatively related to normative commitment (β = -0.76, *p* < 0.01, Model 3 in **Table 2**). We plotted the interaction effect in **Figure 2** based on values plus and minus one standard deviation from the mean of POS (Cohen et al., 2003). The plot shows that the negative relationship between role conflict and normative commitment was more significant when POS was high than when POS was low. Hence, Hypothesis 5b was supported.

Conditional mediation effects of normative commitment on the relationship between role conflict and normative commitment were also tested at different levels of POS. The moderated mediation effect was supported (effect = -0.13, *SE* = 0.04, 95% C.I. = -0.217 to -0.067). To be specific, there was a negative significant indirect effect when POS was high (conditional indirect effect = -0.08, *SE* = 0.03, 95% C.I. = -0.149 to -0.023); and there was a positive significant indirect effect when POS was low (conditional indirect effect = -0.105, *SE* = 0.05, 95% C.I. = 0.017 to 0.214). Thus, the moderated mediation effect of role conflict–normative commitment–helping was supported.

### Discussion

Study 1 explored the negative relationship between role stressors and employees’ helping behavior, the mediating role of normative commitment, and the moderating role of POS in this relationship. Results support that exposure to role ambiguity can decrease employees’ normative commitment and thus helping behavior. In addition, our results revealed that the presence of high POS magnifies rather than weakening the detrimental impact of role conflict on employees’ normative commitment.

Despite the supportive findings, there is an important limitation of Study 1. We constructed the theoretical model based on cognitive dissonance theory to explore the influence of role stressor. However, we only tested the mediating role of attitude change (e.g., normative commitment) as a result of dissonance and failed to monitor employees’ cognition dissonance process. Thus, we conducted Study 2 to address the limitation.

## Study 2

Study 2 is conducted to test whether individuals exposed to a high level of role ambiguity would induce their dissonance, which motivates them to change cognition. By doing so we can verify the cognitive dissonance perspective applied in explaining the role stressor–helping correlation.

### The Mediating Effect of Dissonance

Dissonance is defined as the negative affective state caused by cognition discrepancy (Hinojosa et al., 2017). Previous studies have measured cognitive dissonance as the negative affect and discomfort (e.g., Harmon-Jones, 2000). When exposed to role stressor, employees perceive different role expectations out of their normal repertoire. The discrepancy perception will induce them to experience dissonance. Such uncomfortable feeling can be the motivation for employees to change attitude or belief to the way of their behavior. Most directly, employees’ perceived role stressors are likely to decrease their normative commitment to the organization as stated in Study 1. Thus, according to the cognitive dissonance process, we propose that role stressors induce employees’ dissonance and then induce attitude change toward their organization (e.g., the decrease of normative commitment to the organization). As such, we propose:

Hypothesis 6: Role stressors have a positive relationship *with dissonance.*
Hypothesis 7: Dissonance has a negative relationship with normative commitment.
Hypothesis 8: Dissonance mediates the negative relationship between role stressors and normative commitment.

In Study 2, a scenario experiment was conducted to examine the mediating effect of employees’ cognition dissonance on the role stressor–normative commitment relationship. All procedures were performed in accordance with the 1964 Helsinki Declaration and were approved by the local ethics committee, and written informed consent was obtained from participants.

### Method

#### Participants

One hundred and four Chinese employees were recruited from the Beijing, Hebei, Shandong, and Heilongjiang provinces. We prepared an Internet link to an online survey and e-mailed it to these participants. They were directed to read the scenario and finish the questionnaire online. 57.7% were male (*N* = 60). 89.4% were aged under 29, 8.7% were between 30 and 39 years old, and 1.9% were between 40 and 49 years old. The average tenure of them was 2.87 years (*SD* = 3.15). Regarding education, most participants (*N* = 95) had a bachelor’s degree or higher.

#### Procedure

We adopted a between-subjects experimental design. We recruited 140 working adults as participants in our study through a widely used social network website in China (i.e., Wechat). The participants were randomly assigned to one of two conditions: high role stressors or no role stressor. We sent out the online link to each group (70 participants received the high role stress scenario and 70 participants received non-role stress scenario). 50 participants in the “stressor” group and 65 individuals in the “non-stressor” group finished the survey. We deleted the samples whose answer time is less than 2 min. Finally, we got a sample including 42 participants from the stress group and 62 participants from the non-stress group.

During the experiment process, participants needed to read a story that described an employee experienced with or without role stressors and were told to imagine that they were the employee in the story. Then, we check the manipulation by asking participants to answer questions from the role stressor scale. Finally, we reminded participants to keep imagining themselves as the employee in the story and asked them to report dissonance and normative commitment.

#### Measures

We used the translation–back translation procedure (Brislin, 1980) to ensure the questionnaire’s accuracy.

##### Dissonance

It was assessed with the scale combined discomfort and negative affect assessment (Harmon-Jones, 2000). The assessment of discomfort included the items “uncomfortable,” “uneasy,” and “bothered.” The items “tense,” “distressed,” “irritable,” “nervous,” and “jittery” were used to assess general negative affect. Cronbach’s alpha was 0.79. We did not assess the positive affect because previous studies showed that cognitive dissonance had no influence on it (Elliot and Devine, 1994).

##### Normative commitment

The same scale used in Study 1 was applied. Cronbach’s alpha was 0.76.

##### Control variables

We controlled for employees’ age, gender, education.

### Results

The *t*-test results showed that the manipulation was successful. To be specific, scores of role stressor in the experimental context were significantly higher than those under the control condition (Mrs = 2.54, Mnon-rs = 1.74, *t* = -9.10, df = 102, *p* < 0.01). As expected, the manipulation was successful. As shown in **Table 3**, role stressor was positively correlated with dissonance (*r* = 0.30, *p* < 0.01) and negatively correlated with normative commitment (*r* = -0.38, *p* < 0.01). Thus, Hypothesis 6 was supported. A negative relationship was found between dissonance and normative commitment (*r* = -0.37, *p* < 0.01). Thus, Hypothesis 7 was supported.

**Table 3:** Descriptive statistics, correlations of variables in Study 2.

	Mean	*SD*	1	2	3	4	5
(1) Gender^a^	0.42	0.49					
(2) Age^b^	2.13	0.39	-0.03				
(3) Education^c^	3.29	0.62	-0.09	0.17			
(4) Role stressor^d^	0.60	0.49	-0.09	0.06	0.13		
(5) Dissonance	2.47	0.58	0.01	-0.04	-0.24^∗^	0.30^∗∗^	
(6) Normative commitment	4.02	0.71	0.09	-0.02	0.10	-0.38^∗∗^	-0.37^∗∗^

*n = 104; ^a^Gender: 0 = Male; 1 = Female; ^b^Age: 1 = aged 29 or below; 2 = aged between 30 and 39; 3 = aged between 40 and 49; and 4 = aged 50 or above; ^c^Education: 1 = high school or below; 2 = junior college degree, 3 = bachelor’s degree; and 4 = master’s degree or higher; ^d^Dummy variable: 0 = No role ambiguity; 1 = with role ambiguity; ^∗^*p* < 0.05, ^∗∗^*p* < 0.01*.

As shown in **Table 4**, OLS regression analysis was adopted to test Hypothesis 8. When the mediator (cognitive dissonance) is added (β = -0.28, *p* < 0.001), the effect of role stressor decreases slightly (from -0.38 to -0.30). However, there is still a direct effect. Thus, cognitive dissonance plays a partial mediating effect on the relationship between role stressor and normative commitment. Overall, we can conclude that H8 was supported.

**Table 4:** Results of mediating effect of dissonance in Study 2.

	Dissonance		Normative commitment		
Control variables					
Gender^a^	-0.01	-0.03	0.01	0.13	0.12
Age^b^	0.01	0.01	-0.04	-0.05	-0.05
Education^c^	-0.24^∗^	-0.20^∗^	0.11	0.07	0.01
**Independent variable**					
Role stressor^d^		0.27^∗∗^		-0.38^∗∗∗^	-0.30^∗∗^
**Mediator**					
Dissonance					-0.28^∗∗^
*R*^2^	0.06	0.13	0.02	0.16	0.23^∗∗∗^
ΔR^2^		0.07^∗∗^		0.14^∗∗^	0.07^∗∗^

*n = 104; ^a^Gender: 0 = Male; 1 = Female; ^b^Age: 1 = aged 29 or below; 2 = aged between 30 and 39; 3 = aged between 40 and 49; and 4 = aged 50 or above; ^c^Education: 1 = high school or below; 2 = junior college degree, 3 = bachelor’s degree; and 4 = master’s degree or higher; ^d^Dummy variable: 0 = No role ambiguity; 1 = with role ambiguity; ^∗^*p* < 0.05, ^∗∗^*p* < 0.01, ^∗∗∗^*p* < 0.001*.

### Discussion

In Study 2, we obtained evidence supporting the use of a cognitive dissonance perspective to understand the association between role stressors and normative commitment. We found that dissonance significantly mediated the association between role stressors and normative commitment.

## General Discussion

### Theoretical and Practical Implications

Our research examined the relationship between role stressors and helping behavior combining cognitive dissonance perspective and social exchange theory. Most of the predictions were supported in spite of some unexpected results. To be specific, Study 1 found a significant negative relationship between role ambiguity and normative commitment. However, the negative relationship between role conflict and normative commitment was not supported, which is consistent with prior studies (Jackson and Schuler, 1985; Tubre and Collins, 2000). The reason might be that employees with role conflict have to do a lot of job tasks to satisfy others’ expectation, and the victims can’t identify and distinguish whether the tasks they are doing are helpful for their performance evaluation. Moreover, we proposed competitive assumptions of the moderating pattern of POS. Results supported that POS strengthens the negative effect of role conflict on normative commitment.

Our studies contribute to the literature of role stressors and employees’ helping behavior by combining cognitive dissonance perspective and social exchange theory. Such explanation is meaningful because it indicates that role stressor, an undesirable treatment from the sender on behalf of an organization, can shape one’s perception of cognitive dissonance and thus change their attitude and behaviors. Although previous studies have related subordinates’ perceptions of role stressors to their subsequent commitment (Yousef, 2002; Addae et al., 2008), these studies have all regarded role stressors as a form of work stressors, which can induce employees’ strain. They mainly focus on the relationship-based perspective according to the resource-consuming effect of role stressors. However, we argue that such perspectives cannot reflect the core feature of role stressors. Our studies focus on the cognitive influence of role stressors and explore the relationship between role stressors and helping according to the cognitive dissonance theory and social exchange theory. Such exploration provides a new rationale for explaining the effect of role stressor.

Our research contributes to the job resource literature by suggesting that POS can create a negative effect under stress context. We proposed competitive assumptions of the moderating effect of POS and the findings support that POS strengthens the negative effect of role conflict on normative commitment and thus performs less helping. It supports the function of free choice in the cognition dissonance process and provides a new and rational angel for explaining the moderating effect of POS. According to the cognitive dissonance theory, if there is no choice, dissonance-related attitude change will not occur (Festinger, 2010). When POS is high, employees have more choice and thus increase dissonance perception. It is consistent with the previous findings suggesting that resources can also be a source of strain under some certain contexts (Deelstra et al., 2003; Jain et al., 2013). Thus, our results provide evidence for job resource literature in identifying the contextual situation that resources induce negative results.

Our research also contributes to the literature on helping behavior by exploring the mediating effect of normative commitment. Although previous studies have explored the relationship between role stressors and three forms of commitment, they regard the three forms of commitment as the general attitude. Our research refined the influence on normative commitment (rather than the other kinds of commitment) from the perspective of cognition by considering the feature of normative commitment. As mentioned earlier, the norm perspective has been used as an explanation for the occurrence of helping behavior. However, there are some criticisms of the normative explanations of helping behavior. Schwartz (1973) has called for pointing out the context under which norms will be activated and hence influence behavior. In response to this call, we contend that exposure to role stressors is an important clue to understanding the norm. We extend the work of Kuehn and Al-Busaidi (2002) by exploring the boundary conditions of role stressors in influencing normative commitment and helping behavior. Thus, our research contributes to the existing literature by extending the application of normative explanation to understand helping behavior.

Our findings have several implications for practitioners. First, organizations tend to exploit their employees in today’s highly competitive and rapidly changing environment. Employees have to deal with a lot of role stressors in the workplace. Considering the negative impact of role stressors on helping behavior, organizations should encourage employees to use face-to-face meetings with their supervisors to reduce the occurrence of role ambiguity or role conflict (Walker et al., 1975). They will have more opportunities to understand the supervisors’ expectation if they contact frequently.

Furthermore, managers should be careful to provide organizational support for employees exposed to role stressors, as they are likely to decrease normative commitment and helping behavior. For employees under role ambiguity or conflict, rather than providing them with organizational support, managers may help them find out effective ways to decrease cognitive dissonance and to cope with their discomfort, such as providing training opportunities for them.

### Limitations and Future Research

The current study has several limitations. First, data were all collected from employees; therefore, concerns about common-method bias could be raised. Future studies should consider collecting data from different sources to reduce common-method bias. Second, the research design was cross-sectional. Future research should consider a longitudinal design, whereby measures of role stressors, dissonance, normative commitment, POS, and helping are collected at different time points to ensure the causal effect.

In addition, the Chinese sample might limit the generalizability of our findings (Pellegrini and Scandura, 2008). For example, high-collectivism subordinates may be less likely to decrease their attitude to the organization because they are more tolerant of role stressors in the workplace. As a result, we expect them to exhibit higher normative commitment and adopt higher helping behavior than their low-collectivism counterparts. Future research can investigate whether the relationships identified here can also be replicated in a cross-cultural context.

There are also conceptual limitations in this research. First, we use negative affect to reflect dissonance. Prior studies use different methods to capture dissonance. For example, Sweeney et al. (2000) developed a particular scale of cognitive dissonance after purchase, which includes cognitive and emotional components. Future studies can use different measures to enrich the empirical evidence of cognitive dissonance. Second, we have assumed an implicit mechanism (i.e., freedom of choice) to explain the moderating role of POS. Future research can try to measure autonomy/freedom as a factor to corroborate the current model. Finally, we encourage future studies to explore the effect on other potential outcomes, such as employees’ well-being, performance, or other kinds of OCBs.

## Author Contributions

LZ and YX contributed equally to this paper. BL and LH contributed to the data collection and results section.

## Conflict of Interest Statement

The authors declare that the research was conducted in the absence of any commercial or financial relationships that could be construed as a potential conflict of interest.
